# Shifting the balance: soluble ADAM10 as a potential treatment for Alzheimer's disease

**DOI:** 10.3389/fnagi.2023.1171123

**Published:** 2023-05-17

**Authors:** Ayelet Sarah Hershkovits, Sivan Gelley, Rawad Hanna, Oded Kleifeld, Avidor Shulman, Ayelet Fishman

**Affiliations:** ^1^Department of Biotechnology and Food Engineering Technion-Israel Institute of Technology, Haifa, Israel; ^2^The Interdisciplinary Program for Biotechnology, Technion-Israel Institute of Technology, Haifa, Israel; ^3^Department of Biology Technion-Israel Institute of Technology, Haifa, Israel; ^4^Independent Researcher, Rakefet, Israel

**Keywords:** Alzheimer's disease, ADAM10, amyloid beta, soluble amyloid precursor protein, terminomics, proteomics

## Abstract

**Introduction:**

Accumulation of amyloid β in the brain is regarded as a key initiator of Alzheimer's disease pathology. Processing of the amyloid precursor protein (APP) in the amyloidogenic pathway yields neurotoxic amyloid β species. In the non-amyloidogenic pathway, APP is processed by membrane-bound ADAM10, the main α-secretase in the nervous system. Here we present a new enzymatic approach for the potential treatment of Alzheimer's disease using a soluble form of ADAM10.

**Methods:**

The ability of the soluble ADAM10 to shed overexpressed and endogenous APP was determined with an ADAM10 knockout cell line and a human neuroblastoma cell line, respectively. We further examined its effect on amyloid β aggregation by thioflavin T fluorescence, HPLC, and confocal microscopy. Using N-terminal and C-terminal enrichment proteomic approaches, we identified soluble ADAM10 substrates. Finally, a truncated soluble ADAM10, based on the catalytic domain, was expressed in *Escherichia coli* for the first time, and its activity was evaluated.

**Results:**

The soluble enzyme hydrolyzes APP and releases the neuroprotective soluble APPα when exogenously added to cell cultures. The soluble ADAM10 inhibits the formation and aggregation of characteristic amyloid β extracellular neuronal aggregates. The proteomic investigation identified new and verified known substrates, such as VGF and N-cadherin, respectively. The truncated variant also exhibited α-secretase capacity as shown with a specific ADAM10 fluorescent substrate in addition to shedding overexpressed and endogenous APP.

**Discussion:**

Our *in vitro* study demonstrates that exogenous treatment with a soluble variant of ADAM10 would shift the balance toward the non-amyloidogenic pathway, thus utilizing its natural neuroprotective effect and inhibiting the main neurotoxic amyloid β species. The potential of such a treatment for Alzheimer's disease needs to be further evaluated *in vivo*.

## 1. Introduction

Alzheimer's disease (AD) is an irreversible neurodegenerative disease of the brain and the most common form of dementia, currently accounting for 60%−80% of all cases. Over 55 million people have been diagnosed with dementia worldwide, a number expected to increase to ~80 million by 2030 (Alzheimer's Disease International, 2021). AD is characterized by a gradual decline in cognitive abilities such as decreased memory and learning ability, language impairment, and the inability to perform basic daily functions and social interactions (Scheltens et al., 2021; Alzheimer's Association, 2022) Prior to the emergence of the clinical cognitive symptoms of the disease, the patient presents preclinical biological signs that typically occur over two decades before the clinical symptoms (Long and Holtzman, 2019). The hallmark neuropathologies of AD are the accumulation of extracellular amyloid β (Aβ) plaques (Thal et al., 2002) and the formation of intracellular neurofibrillary tangles (tau proteins) in the neurons (Braak and Braak, 1991), both causing degeneration and death of nerve cells in the brain (Bloom, 2014).

According to the Aβ hypothesis, the accumulation of the neurotoxic Aβ initiates the downstream processes, leading to AD pathologies such as tau aggregation, loss of neurons, and synaptic connections (Hardy and Higgins, 1992; Selkoe and Hardy, 2016; Karran and de Strooper, 2022). The Aβ monomer is a cleavage product of the amyloid precursor protein (APP) in the amyloidogenic processing pathway (Müller et al., 2017). β-secretase mediates the first proteolysis step, cleaving the transmembrane protein APP, followed by the γ-secretase releasing the Aβ peptide, generally consisting of 38–42 residues (Nunan and Small, 2000; Masters and Selkoe, 2012). The longer hydrophobic Aβ peptide containing 42 residues (Aβ42) is prone to aggregation leading to the formation of the neurotoxic Aβ oligomers and fibrils present in the amyloid plaques (Ahmed et al., 2010; Masters and Selkoe, 2012). However, in the parallel non-amyloidogenic pathway, APP undergoes cleavage in the Aβ sequence by an α-secretase thus preventing the formation of the Aβ peptide and releasing the neuroprotective soluble APPα (sAPPα) (Furukawa et al., 2002; Richter et al., 2018). The main α-secretase in the nervous system, and the enzyme well-known to be involved in the non-amyloidogenic pathway, is a multi-domain transmembrane protein known as a disintegrin and metalloprotease domain 10 (ADAM10) (Lammich et al., 1999; Kuhn et al., 2010). In the extracellular part of the protein, the pro-domain acts as a specific inhibitor of the metalloprotease activity, thus following its removal in the secretory pathway, the protein becomes catalytically active (Moss et al., 2007). The metalloprotease catalytic domain is followed by a disintegrin domain, a cysteine-rich domain, a transmembrane domain, and a cytoplasmic tail (Reiss and Saftig, 2009). The disintegrin and cysteine-rich domains have been shown to be involved in substrate recognition and the autoregulation of enzymatic activity (Janes et al., 2005; Seegar et al., 2017).

ADAM10 has multiple neuronal substrates and functions and is involved in embryonic brain development, synaptic function, and adhesion (Reiss and Saftig, 2009; Saftig and Lichtenthaler, 2015; Kuhn et al., 2016). Dysregulation of ADAM10 is also associated with different diseases such as AD, prion disease, and different types of cancer (Pruessmeyer and Ludwig, 2009; Endres and Fahrenholz, 2012; Guo et al., 2012; Altmeppen et al., 2015; Mullooly et al., 2015). ADAM10 as a therapeutic target in AD is thought to be beneficial for disease treatment as it initiates the non-amyloidogenic pathway and could prevent the downstream effects of AD (Endres and Fahrenholz, 2010; Lichtenthaler, 2011; Marcello et al., 2017; Lichtenthaler et al., 2022). Previous research demonstrated that overexpression of membrane-bound ADAM10 prevented Aβ plaque formation and neuronal defects in mice (Postina et al., 2004). A study assessing the levels of ADAM10 in the platelets and cerebral spinal fluid of AD patients found that the ADAM10 levels are reduced as compared to non-AD controls (Colciaghi et al., 2002, 2004; Sogorb-Esteve et al., 2018). A pilot clinical study showed that the pharmacological activation of endogenous ADAM10 resulted in enhanced APP cleavage with no adverse effects in AD patients (Endres et al., 2014). Furthermore, proteome analysis of patient samples revealed that the activation of ADAM10 may be safer than first perceived with regard to its other substrates apart from APP (Brummer et al., 2019). More recently, an *in vivo* study evaluated a peptide capable of restoring the localization and upregulation of ADAM10 activity at the early stages of the disease, hence rescuing cognitive defects, further supporting the therapeutic potential of ADAM10 in AD (Musardo et al., 2022).

While most developed AD drugs aim to inhibit β- and γ-secretases, prevent Aβ aggregation, or utilize anti-Aβ antibodies to promote Aβ aggregate clearance from the brain (Cummings et al., 2022; Karran and de Strooper, 2022), we propose a new enzymatic approach of using a novel soluble form of ADAM10 for the treatment of AD. The main objective of this research was to develop a new treatment for AD using a truncated version of ADAM10, based on the catalytic metalloprotease domain. This tactic was evaluated using *in vitro* studies. First, we examined the ability of a soluble ADAM10 consisting of multiple domains, to shed APP. Specifically, we demonstrated that the soluble enzyme is capable of shedding overexpressed APP and endogenous APP from neuronal cells resulting in an increase of sAPPα and a decrease in the neurotoxic Aβ42. Furthermore, we examined the effect of the soluble variant on Aβ42 aggregation. The soluble ADAM10 hydrolyzes Aβ42 and inhibits its aggregation process in neuronal cells. Considering the large array of membrane-bound ADAM10 substrates and their pathological effects, we began to evaluate potential substrates of the soluble enzyme using N-terminal and C-terminal enrichment proteomics approaches. Finally, we expressed a truncated variant of the soluble ADAM10 consisting solely of the metalloprotease catalytic domain in *Escherichia coli*. We found that the truncated soluble enzyme is active and capable of significantly shedding APP and releasing sAPPα above its endogenous level. This AD treatment approach shifts APP shedding toward the non-amyloidogenic pathway, inhibiting Aβ production, increasing production of sAPPα, and degrading existent Aβ aggregates.

## 2. Materials and methods

### 2.1. Cell culture

Human embryonic kidney 293T (HEK293T) ADAM10 knockout cells (Brummer et al., 2018) (provided by Dr. Michael G. Tomlinson, University of Birmingham) were cultured in Dulbecco's Modified Eagle's Medium (DMEM high glucose, Sigma) supplemented with 10% heat-inactivated fetal bovine serum (FBS), 1% penicillin–streptomycin, 1% amphotericin B, and 1% non-essential amino acids (all from Biological Industries, Israel).

Neuroblastoma SH-SY5Y cells (ATCC, CRL 2266) were cultured in DMEM:F12 (1:1) supplemented with 10% heat-inactivated FBS, 1% penicillin–streptomycin, 1% amphotericin, and 1% non-essential amino acids. For the induction of neuronal differentiation, cells were seeded and allowed to adhere for 24 h. Next, the media was replaced, and cells were cultured in growth media containing 1% heat-inactivated FBS and 10 μM all-*trans* retinoic acid (Sigma) for 4 days (adapted from Encinas et al., 2002).

All cultures were maintained in a humidified 37°C incubator with a 5% CO_2_ atmosphere.

### 2.2. APP shedding of alkaline phosphatase fusion protein

To determine the shedding of APP by soluble ADAM10, an APP-secreted embryonic alkaline phosphatase (SEAP) fusion protein was utilized as previously described (Lichtenthaler et al., 2003). HEK293T ADAM10 knockout cells were transiently transfected with a pEAK12-SEAP/APP plasmid (provided by Prof. Stefan Lichtenthaler, German Center for Neurodegenerative Diseases), using CalFectin DNA Transfection Reagent (SignaGen) according to the manufacturer's manual. The cells were dissociated 18-h post-transfection, washed with PBS, and re-seeded in a 48-well plate with complete media at 1.5 × 10^5^ cells per well. After 24 h, the cells were washed with PBS, treated with commercial soluble recombinant human ADAM10 (#S936-AD-020, R&D Systems) in treatment media (DMEM with high glucose, without glutamine or phenol red, supplemented with 1% penicillin–streptomycin, 1% amphotericin B, 1% non-essential amino acids, and 1% L-glutamine), and incubated for 48 h (37°C, 5% CO_2_) before alkaline phosphatase activity in the media was determined. First, the media was collected into Eppendorf tubes and centrifuged (250 g, RT, 5 min) to remove cellular debris, followed by heat treatment of the supernatant (65°C, 30 min) to inactivate endogenous alkaline phosphatase activity. Next, 100 μl of the samples and reaction buffer (1 mg/ml of pNPP in 0.1 M glycine, 1 mM MgCl_2_, 1 mM ZnCl_2_, pH 10.4) were equilibrated separately in a 96-well plate at 37°C for 10 min. The assay was initiated by the addition of 100 μl of equilibrated reaction buffer to the samples, and absorption was measured immediately. Absorbance at a wavelength of 405 nm was measured in a Synergy H1 microplate reader (BioTek) at 37°C with 30-s intervals for 30 min. The hydrolysis rate of pNP-phosphate was defined as the linear slope (*R*^2^ > 0.99) of absorbance vs. time over 20-time points and expressed as OD405 nm/min. Activity in treated wells was expressed as the relative increase in activity slope compared to untreated wells.

### 2.3. APP shedding of the fluorescent fusion protein

Amyloid precursor protein shedding was also assessed by the cleavage of an APP dual fluorescent fusion protein (Parenti et al., 2017). The APP fusion plasmid, mCherry-APP-mGFP (provided by Dr. Martino Calamai, Italian National Research Council), consists of a green fluorescent protein (mGFP) at the C-terminal and a red fluorescent protein (mCherry) at the N-terminal of the APP 695 isoform. The plasmid was transiently transfected to HEK293T ADAM10 knockout cells using CalFectin DNA Transfection Reagent. The cells were dissociated 18-h post-transfection, washed with PBS, and re-seeded in a 48-well plate with complete media at 10^5^ cells per well. After 24 h, the cells were washed with PBS, treated with commercial soluble recombinant human ADAM10 in treatment media, and incubated for 48 h. Un-transfected cells were counted and plated in a similar manner as “blank” controls for the reduction of background fluorescence. Following incubation, the media was collected, centrifuged, and the media fluorescence was measured in a 96-well half-area black plate (excitation 550 nm, emission 610 nm) using a Synergy H1 microplate reader for 10 reads per well. Soluble ADAM10 activity was expressed as the fold increase in averaged media fluorescence compared to the untreated controls.

### 2.4. Detection of APP shedding by flow cytometry

The mCherry-APP-mGFP plasmid was also utilized to determine the shedding of APP by flow cytometry. For flow cytometry compensation, two mCherry-APP-mGFP mutant plasmids were prepared. Specifically, a point mutation at Met72Lys rendered the mCherry non-fluorescent (Gomes et al., 2016) and a point mutation at Gly67Ala resulted in a non-fluorescent GFP, as previously described (Wielgus-Kutrowska et al., 2007). For flow cytometry analysis, HEK293T ADAM10 knockout cells were transfected with the mCherry-APP-mGFP, the mCherry mutant, or the GFP mutant plasmid using CalFectin DNA Transfection Reagent. Following 18 h post transfection, the cells were dissociated, washed with PBS, and re-seeded in a 12-well plate at 2 × 10^5^ cells per well with complete media. Cells were allowed to adhere for 24 h after which they were washed with PBS and treated with commercial soluble recombinant human ADAM10 in treatment media and incubated for 48 h. Next, the cells were extracted using a non-enzymatic cell dissociation solution (Gibco, Thermo Fisher), centrifuged, washed twice in PBS, and re-suspended in PBS for flow cytometry analysis. Flow cytometry was performed with a Cytek^®^ Aurora, and the results were analyzed using FCS Express (De Novo Software). First, cellular debris was excluded, and the main cell population was determined by gating non-transfected cells on the side scatter area vs. forward scatter area graph. Single cells were included and doublets were excluded from the quantification by gating on the forward scatter area vs. forward scatter width. Next, the autofluorescence of the cells was marked. Finally, the transfected cells were analyzed with a fluorescence scatter plot of GFP fluorescence vs. mCherry fluorescence. Double positive cells (i.e., GFP and mCherry fluorescence) were used for the analysis, and the mCherry mean fluorescence intensity was determined. The results are presented as relative mCherry mean fluorescence intensity.

### 2.5. Detection of endogenous APP shedding by Western blot

Endogenous shedding of APP from differentiated SH-SY5Y cells was assessed by Western blot analysis of sAPPα in culture media post-treatment. Briefly, 0.5 × 10^6^ SH-SY5Y cells per well were seeded for differentiation in a six-well plate. Following the differentiation protocol, the cells were washed with PBS once and the media was replaced with commercial soluble recombinant human ADAM10 in neuronal treatment media [DMEM:F12 (1:1) supplemented with 1% penicillin–streptomycin, 1% amphotericin B, and 1% non-essential amino acids] and incubated at 37°C, 5% CO_2_. Following 48 h post-treatment, the media was collected, centrifuged (250 g, 4°C, 5 min) to remove debris, followed by concentration of the supernatant with a centrifugal filtration unit (AmiconUltra, 10 kDa cutoff) and kept on ice. The cells were also collected, centrifuged, and lysed in ice-cold lysis buffer [1% Triton, 20 mM Tris pH 8, 37 mM NaCl, 10% glycerol with a protease inhibitor cocktail (cOmplete, Sigma)], placed on ice for 10 min and centrifuged (10,000 g, 4°C, 10 min). The total protein concentration of the supernatant was determined using the Bradford method. Next, media sample normalization was achieved by preparing samples standardized to total lysate protein concentration as previously described (Levites et al., 2003; Adlerz et al., 2007; Colombo et al., 2013). SDS-PAGE sample buffer X4 was then added to the concentrated media, and samples were boiled before separation on an 8% SDS–polyacrylamide gel and transferred to a nitrocellulose membrane. The membrane was then blocked with 5% non-fat dry milk in TBST buffer, incubated with the primary antibody (mouse anti-sAPPα), specific to Aβ1-16 which binds to sAPPα epitope (Obregon et al., 2012) (6E10, 1:2000, BioLegend, #80300) for 2 h at RT followed by three washes with TBST and incubation with sheep anti-mouse HRP-conjugated secondary antibody (1:10,000, Amersham, #NA931) for 1 hr at RT. Finally, the membrane was washed and visualized with Immobilon Crescendo Western HRP chemiluminescence substrate (Thermo Fisher) using the FUSION FX Spectra imaging system. The relative intensity of the bands was integrated by the Evolution Cap Edge software supplied with the FUSION FX imaging device. Activity in treated wells was expressed as the relative increase in band intensity compared to untreated wells.

### 2.6. Aβ42 quantification by ELISA

Endogenous Aβ42 peptide in the media of differentiated SH-SY5Y cells following treatment was quantified using the Human Aβ42 ELISA kit according to the manufacturer's instructions (MyBioSource, #MBS268504). The cells were prepared and treated exactly as described for the Western blot shedding experiment. Following 48 h post treatment, the media was collected and placed on ice while the cells were lysed, and the total protein in the lysate was determined using the Bradford method. The media was concentrated by lyophilization for 24 h. The volume of concentrated media was normalized to the cell amount based on the protein concentration of the cell lysate. The lyophilized samples were re-suspended in the sample buffer supplied with the kit. Aβ42 in the concentrated media was quantified using the ELISA kit. Aβ42 levels are represented as relative to the media from untreated control cells.

### 2.7. Preparation of monomeric Aβ42

Monomeric Aβ42 was prepared by dissolving Aβ42 (#052487, GL Biochem Ltd, Shanghai, China) in 1,1,1,3,3,3-hexafluoro-2-propanol (HFIP, Sigma) (Stine et al., 2003). Aβ42 was dissolved in ice-cold HFIP to 1 mg/ml and incubated for 1 h at room temperature. Next, the solution was aliquoted, and the HFIP evaporated with a gentle N_2_ stream for 40 min, followed by concentration with a SpeedVac vacuum concentrator for 1 h. The resulting peptide films were stored at −20°C until use. Prior to each experiment, Aβ42 concentration was quantified using the bicinchoninic acid assay (BCA) with a standard curve of Aβ40 (#051516, GL Biochem Ltd, Shanghai, China), as previously described (Jan et al., 2010).

### 2.8. Thioflavin T aggregation assay

Monomeric Aβ42 films were thawed on ice and re-suspended in Thioflavin T (ThT) assay buffer (20 mM sodium phosphate buffer, pH 7.4, 0.15 M NaCl), sonicated shortly, and centrifuged (11,000 *g*, 15 min, 4°C). The protein concentration of the supernatant was determined using the BCA assay, as described above, and kept on ice until use. For the aggregation assay, 4 μM of Aβ42 with or without treatments [e.g., commercially soluble recombinant human ADAM10 or plasmin (#P1867, Sigma)] was incubated in the ThT assay buffer in the presence of 10 μM ThT (Sigma), and fluorescence was measured immediately with excitation and emission wavelengths of 440 and 485 nm, respectively. The assays were performed in a clear bottom, black 96-well plate, sealed to prevent evaporation, at 37°C with continuous shaking in a Synergy H1 microplate reader.

### 2.9. Proteolytic activity analysis by HPLC

High-performance liquid chromatography (HPLC) was carried out to evaluate the ability of soluble ADAM10 to cleave Aβ42. Monomeric Aβ42 was prepared and quantified prior to the experiments as described for the ThT aggregation assay above. Next, 40 μM of Aβ42 with or without treatments, commercially soluble recombinant human ADAM10 or plasmin, was incubated at 37°C with constant shaking (150 rpm). Following incubation, the samples were centrifuged (11,000 g, 10 min, 4°C) and the supernatant was analyzed by HPLC. The formation of Aβ16 was determined to follow Aβ42 cleavage on a Dionex UltiMate 3000 HPLC (Thermo Scientific) equipped with a diode array detector. Separation was performed with a C4 column (DAISOGEL SP-300-5μ-C4-BIO column, 300 Å, 250 mm × 4.6 mm, 5 μm, Dr. Maisch GmbH, Germany), and data were analyzed using Thermo Scientific Dionex Chromeleon software version 7.1. Mobile phase A was 0.1% trifluoroacetic acid (TFA) in water, and mobile phase B was 0.1% TFA in acetonitrile at a flow rate of 1 ml/min. The column was equilibrated in 90% mobile phase A and 10% mobile phase B, followed by the injection of samples (20 μl). The gradient elution was performed from 10 to 25% mobile phase B over 5 min, followed by 25 to 70% mobile phase B over 5 min, and holding at 70% mobile phase B for 10 min. Detection was carried out at 220 nm. The concentration of the Aβ16 product was determined by a standard curve prepared with synthetic Aβ16 (Genemed Synthesis).

### 2.10. Aβ42 aggregation analysis by confocal microscopy

To assess the effect of soluble ADAM10 on the formation of Aβ42 aggregates, confocal scanning microscopy was utilized. SH-SY5Y cells were seeded in 8-well μ-slides (ibidi, #80826) at 50,000 cells per well, followed by the differentiation protocol. Monomeric Aβ42 films were thawed on ice and re-suspended in PBS before being briefly sonicated and centrifuged (11,000 *g*, 15 min, 4°C). The protein concentration in the supernatant was determined using the BCA assay and kept on ice until use. Following differentiation, the cells were washed once with PBS, and 20 μM of monomeric Aβ42 was added to the cells with or without treatment in neuronal treatment media. Following 24 h post-treatment, the cells were fixed with 4% paraformaldehyde for 20 min and washed with PBS. The cells were permeabilized with 1% Triton X-100 for 10 min and washed with PBS, before blocking with 5% FBS in PBS for 1 h at RT. Aβ42 aggregates were stained overnight with a mouse anti-Aβ42 primary antibody (6E10, 1:500, BioLegend, #80300) in antibody buffer (3% FBS, 0.1% TritonX100) at 4°C. Following staining, the cells were washed with PBS and incubated with donkey anti-mouse CF555 (1:500, Biotium, #20037), DAPI (1:10,000, Biotium, #40043), and Phalloidin-FITC (1:100, Sigma, P5282) in antibody buffer for 1 h, at RT in the dark. Finally, the cells were washed and visualized with the Zeiss LSM700 inverted laser scanning confocal microscope with an X40 oil immersion objective. To determine the Aβ42 aggregates per cell, the analysis included three-dimensional visualization of the z-stack acquisitions and quantification of aggregates and cell volume with Imaris software as follows. DAPI staining (blue channel) was utilized to calculate the total and single cell volume, FITC staining of F-actin (green channel) to visualize the periphery of cells, and Aβ42 aggregates (red channel) analysis to quantify the total aggregate volume. Levels of aggregates per cell are represented as relative to the untreated controls.

### 2.11. Proteomic analysis

A proteomic approach was applied to evaluate the potential soluble ADAM10 substrates of differentiated neuronal cells. SH-SY5Y cells were seeded in a 10 cm culture dish at 8 × 10^6^ cells per plate, followed by the differentiation protocol. Once differentiated, the cells were washed three times with PBS followed by treatment with or without 10 μg/ml of commercial sADAM10 in fresh neuronal media, without serum and phenol red (supplemented with 1% penicillin–streptomycin, 1% amphotericin B, and 1% non-essential amino acids). Following 48 h post-treatment, the media was collected and prepared for proteomic analysis. The media was centrifuged twice (1,800 g, 5 min, 4°C), filtered (45 μm), and a protease inhibitor was added. The secretome was concentrated at 4°C with a centrifugal filter (AmiconUltra, 3 kDa cutoff), in which buffer exchange to 100 mM HEPES pH 7 was performed. Protein concentration in the retained volume was determined using the BCA assay. The samples were then subjected to a denaturation, reduction, and alkylation process. The control samples were labeled with light formaldehyde (C^12^H_2_), and the treated samples were labeled with heavy formaldehyde (C^13^D_2_). Finally, the samples were divided, and half of each sample was taken to either N-terminal enrichment based on HYTANE (Chen et al., 2016; Weng et al., 2019) method using the protocol described in Hanna et al. (2023) or C-terminal enrichment. Following enrichment and desalting, the samples were analyzed by tandem mass spectrometry using Thermo Q-Exactive Orbitrap HF coupled with Easy nano-LC 1000 capillary HPLC. Enriched Terminal peptides were resolved in a homemade reverse phase capillary 30 cm long and 75 μm diameter, packed with 3.5 μm silica using ReproSil-Pur C18-AQ resin (Dr. Maisch GmbH). The elution was carried out with a 120 min linear gradient of 5%−28% acetonitrile (in 0.1% formic acid), followed by a 15-min wash with 95% acetonitrile (in 0.1% formic acid), with all flow rates set to 0.15 μl/min. MS was performed in a data-dependent acquisition mode for positive ions in an *m*/*z* range of 300–1,800, with 120,000 resolution for MS1 and 15,000 resolution for MS2. Ions were fragmented using high-energy collisional dissociation (HCD) with a normalized collision energy (NCE) of 27. Data analysis was performed using the Trans-Proteomic Pipeline (version 6.1) (Deutsch et al., 2010). A peptide search was carried out using COMET (version 2020_01 rev1) (Eng et al., 2013), and peptide scoring was performed using PeptideProphet (Keller et al., 2002) at a false discovery rate of 1%. The peak area ratio was calculated using XPRESS, and the peptides ratio was normalized by subtracting the ratio's median of every sample. All data were searched against UniProt *homo sapiens* proteome containing 73248 sequences (downloaded June 2019). Venn diagram was generated with DeepVenn (https://arxiv.org/abs/2210.04597v1) and the cleavage site sequence logo with IceLogo (Colaert et al., 2009).

Validation of selected substrates was achieved by Western blot analysis. The cells were prepared and treated exactly as described for the Western blot shedding experiment. Prepared samples were separated on a 10% SDS–polyacrylamide gel followed by transfer to a nitrocellulose membrane. Membranes were then blocked with 5% non-fat dry milk in TBST buffer for 2 h and incubated with a primary antibody overnight at 4°C. The following primary antibodies (all purchased from Abcam) in 3% BSA in TBST were used: ab76011 for cadherin-2 (1:1,000) and ab235444 for sulfhydryl oxidase 1 (1:1,000). Next, the membranes were washed with TBST followed by incubation with the HRP-conjugated secondary antibody (goat anti-rabbit HRP, ab97051, 1:5,000) for 2 h. Finally, the membranes were washed again and visualized with an HRP substrate using the FUSION FX Spectra imaging system. Each lane is a separate biological replicate.

### 2.12. Protein expression and purification of truncated sADAM10

A truncated human ADAM10 variant containing a pelB secretion signal at the N-terminal, the human ADAM10 metalloprotease catalytic domain, and a C-terminal 6xHistidine-tag was cloned in a pET22b vector and designated pET22b-ADAM10_MP, resulting in a soluble ADAM10 catalytic domain expressed to the periplasm. Following sequence confirmation, the truncated ADAM10 plasmids were electro-transformed into *E. coli* BL21 (DE3) cells for expression. Cultures were grown for 24 h in Luria–Bertani (LB) medium supplemented with 100 μg/ml of ampicillin at 20°C and harvested by centrifugation (4,500 *g*, 10 min, RT). Cells were re-suspended in binding buffer (50 mM Tris–HCl, pH 8) and disrupted by osmotic shock (20 mM Tris–HCl pH8.0, 2.5 mM EDTA and 20% sucrose) or sonication (Vibra-Cell VCX750 with a CV33 transducer and SM0401tip, Sonics & Materials Inc., Newtown, CT). Cell debris was removed by centrifugation (16,000 g, 20 min, 4°C, twice). The soluble cell extract was applied to a 2.5 ml WorkBeads 40Q ion exchange resin pre-equilibrated with the binding buffer using an AKTA prime plus FPLC system (GE Healthcare Bio-Sciences AB). The flow-through (containing ADAM10_MP) was collected, and the host cell proteins were eluted in a single step using the same buffer supplemented with 1M NaCl. The flow-through was reloaded to the anion exchange column and the flow-through (still containing ADAM10 MP) was collected again. Finally, the enriched flow-through was loaded on a nickel affinity column (HisTrap HP 5mL, #17524802, Cytiva) pre-equilibrated with a binding buffer. Following a washing step with the same buffer containing 50 mM imidazole, ADAM10 MP was eluted in 50 mM Tris–HCl pH 8 with 500 mM imidazole. The eluted fractions were dialyzed against the binding buffer, concentrated X20 (AmiconUltra, 10 kDa cutoff), and evaluated by SDS-PAGE for purity and identity with a mouse anti–His-tag HRP antibody (R&D systems, #MAB050H). The same process was applied to the purification of the control plasmid, pET22b, without an insert.

### 2.13. Activity measurement by fluorescent peptide hydrolysis

The activity of the truncated sADAM10 and its control, expressed from pET22b-ADAM10_MP and pET22b, respectively, was determined utilizing a fluorescent ADAM10 peptide substrate as previously described (Seegar et al., 2017). The fluorescently labeled peptide Mca-PLAQAV-Dpa-RSSSR-NH2 (# ES003, R&D Systems) and samples were first diluted in the reaction buffer [25 mM Tris–HCl, pH 9.0, containing 8 μM ZnCl_2_, and 0.005% (w/v) Brij-35] and equilibrated separately in a 96-well half-area black plate at 37°C for 5 min. Next, the cleavage assay was initiated by mixing 40 μM substrate with the enzyme samples. Fluorescence was measured immediately at 37°C with excitation and emission wavelengths of 320 and 405 nm, respectively. The rate of hydrolysis was defined as the maximal linear slope (*R*^2^ > 0.95) of fluorescence versus time and expressed as relative fluorescence units (RFUs)/min. The activity was expressed as the relative increase in activity slope compared to the control wells.

### 2.14. Statistical analysis

Statistical analysis for experiments was performed using a two-tail distribution *t*-test with unequal variances with Microsoft Excel software.

## 3. Results

### 3.1. Soluble ADAM10 is capable of shedding overexpressed APP from the cell membrane

**Figure 1:**
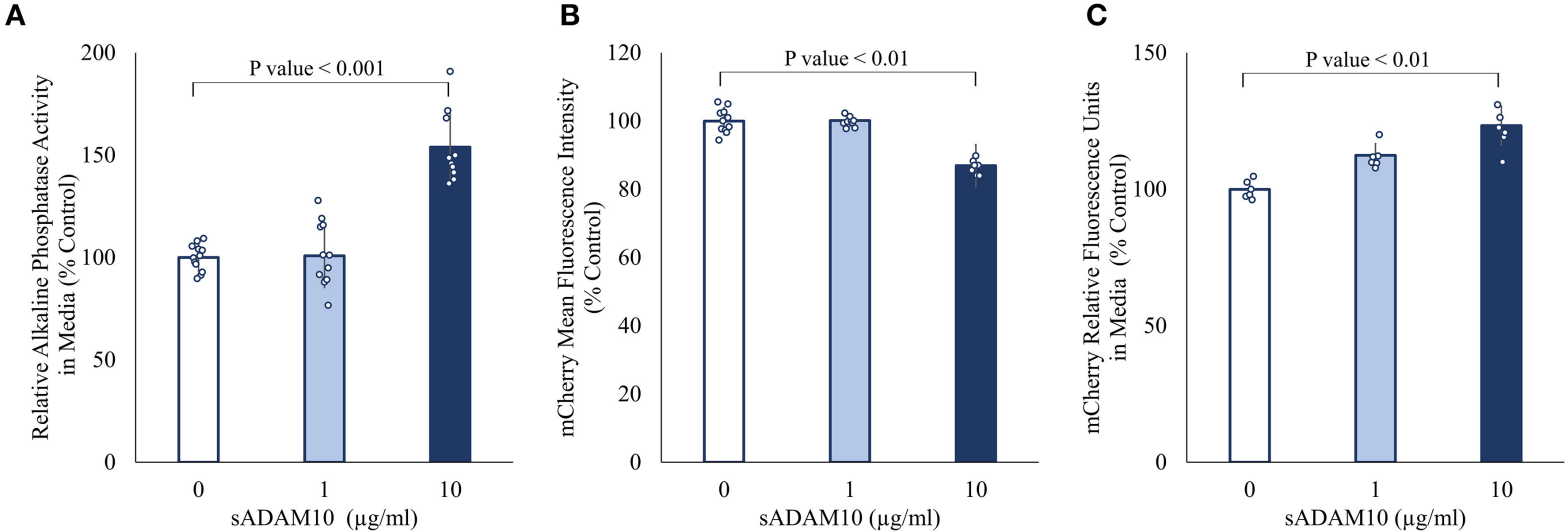
Effect of sADAM10 treatment on APP shedding in HEK293T ADAM10 knockout cells overexpressing APP. **(A)** Relative alkaline phosphatase activity in media 48-h post-treatment of SEAP-APP transfected cells. **(B)** Flow cytometry analysis of fluorescent APP transfected cells 48-h post-treatment. **(C)** Analysis of soluble APP in media following the shedding of fluorescent APP. Data are from three independent experiments performed in duplicate.

Surface-presented APP of neuronal cells was endogenously shed by membrane-bound ADAM10 (Lammich et al., 1999; Kuhn et al., 2010). Nonetheless, the capacity of a soluble form of ADAM10 to hydrolyze APP from the membrane has not been investigated. Initially, the ability of the commercially available soluble ADAM10 (sADAM10), consisting of the metalloprotease catalytic domain, a disintegrin domain, and a cysteine-rich domain, to shed overexpressed APP from the cell surface was determined with two APP fusion variants (Figure 1). To exclude endogenous ADAM10 shedding, HEK293T ADAM10 knockout cells were utilized (Brummer et al., 2018). The cells were transiently transfected with a plasmid encoding APP linked to alkaline phosphatase at the N-terminal (Lichtenthaler et al., 2003), and the shedding was evaluated by the relative enzymatic activity of the alkaline phosphatase in the media following treatment (Figure 1A). A 50% increase in phosphatase activity was measured when cells were treated with a higher concentration of sADAM10. Enzymatic shedding was further assessed with an APP fluorescent fusion protein, consisting of a red fluorescent protein (mCherry) at the N-terminal (Parenti et al., 2017). Similarly, the knockout cells were transiently transfected, followed by treatment with sADAM10. APP shedding from the cell surface was measured by flow cytometry, indicating a 20% decrease in the fluorescence intensity of cell-bound mCherry (Figure 1B). A correlative increase in fluorescence in the media following treatment further supports the ability of the protease to shed APP from cell membranes (Figure 1C).

### 3.2. Soluble ADAM10 sheds endogenous APP from differentiated SH-SY5Y cells

**Figure 2:**
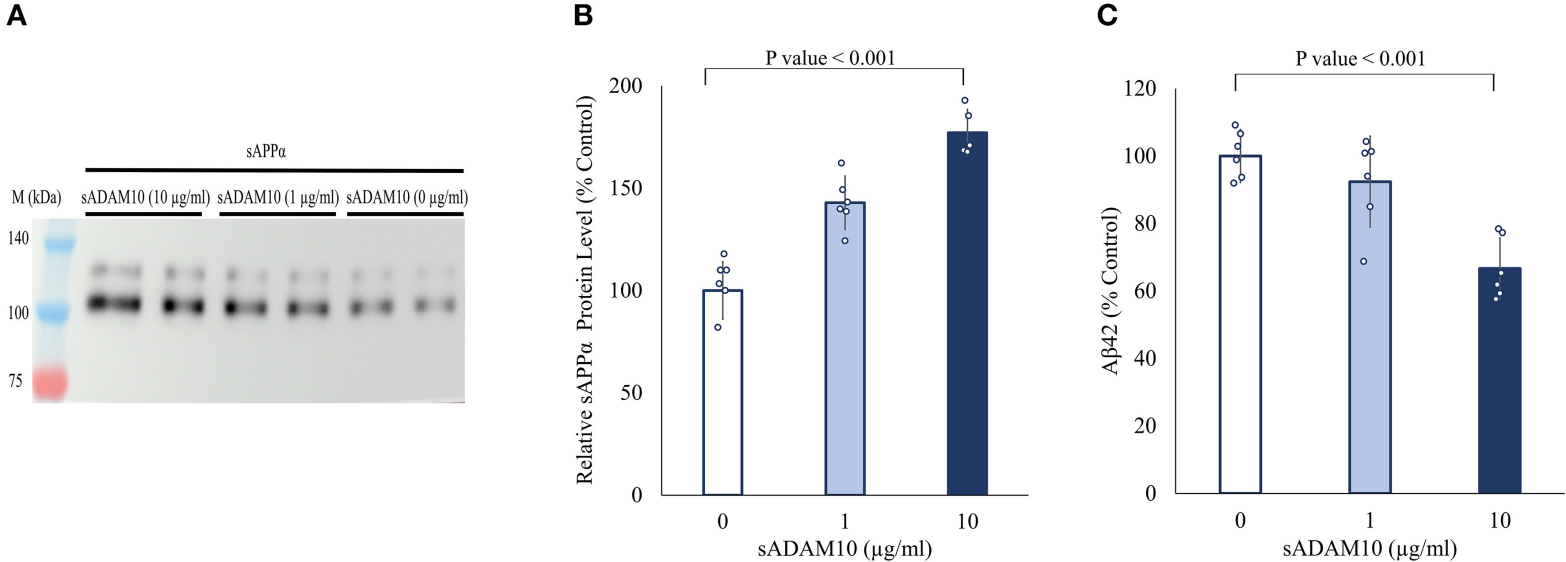
Effect of sADAM10 treatment on endogenous APP shedding in differentiated SH-SY5Y cells. **(A)** Representative Western blot analysis image of endogenous sAPPα in the media of treated cells 48-h post-treatment. **(B)** Western blot quantification of relative sAPPα levels. Data are from three independent experiments performed in duplicate. **(C)** Aβ42 levels in the media of treated cells 48-h post-treatment measured by ELISA. Data are from two independent experiments performed in duplicate.

Next, the ability of sADAM10 to shed endogenous APP was determined by the release of the ADAM10 APP cleavage product, sAPPα. For this aim, differentiated SH-SY5Y cells were treated with sADAM10, and the relative sAPPα levels in the media were measured by Western blot analysis utilizing an antibody specific to the sAPPα epitope (Obregon et al., 2012). An 80% increase (*p* < 0.001) of sAPPα in the media was measured compared to the control when cells were treated with 10 μg/ml of enzyme detected by Western blot analysis (Figures 2A, B). These results suggest an increase in APP cleavage via the non-amyloidogenic pathway. Additionally, a 33% decrease in Aβ42 concentration in the media of treated neuronal cells was measured using an ELISA assay (Figure 2C), indicative of lower shedding of APP from the membrane via the amyloidogenic pathway. However, it could also indicate the hydrolysis of the Aβ42 peptide by the sADAM10 or in fact both, as per the higher levels of sAPPα in the media.

### 3.3. Treatment with the soluble secretase inhibits Aβ42 aggregation

**Figure 3:**
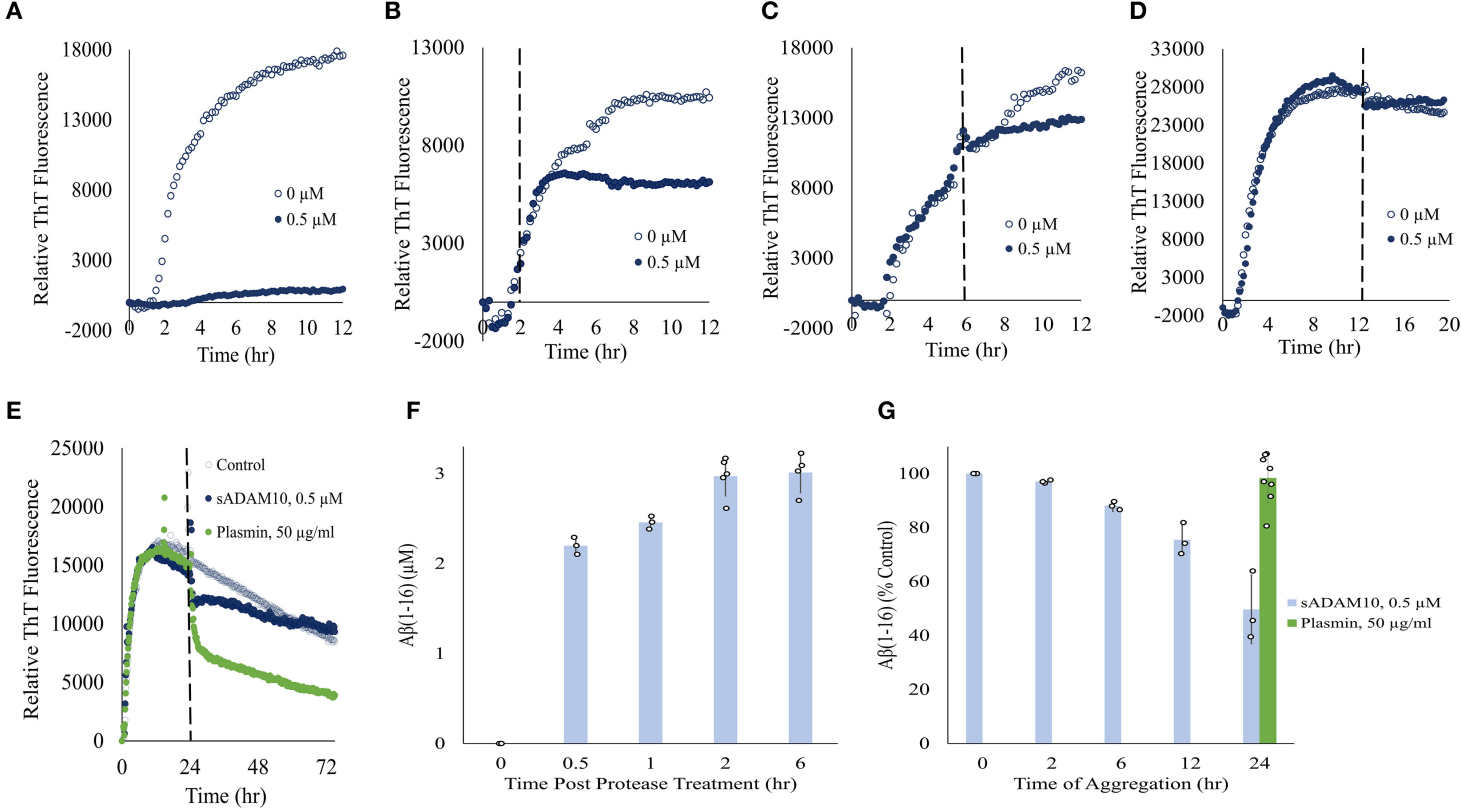
Effect of sADAM10 treatment on Aβ42 aggregation and degradation. **(A–D)** Aβ42 aggregation was monitored by ThT fluorescence with sADAM10 treatment (0.5 μM) at different time points: 0, 2, 6, and 12 h, respectively. **(E)** Aβ42 aggregation was monitored by ThT fluorescence with sADAM10 or plasmin treatment at 24 h. The dashed line represents the time point of treatment. Data are from two independent experiments performed in duplicate. Representative graphs from each concentration are shown. **(F)** HPLC analysis of Aβ16 product following the incubation of monomeric Aβ42 with sADAM10. **(G)** HPLC analysis of Aβ16 product following incubation of aggregated Aβ42 with sADAM10. Data are from three independent experiments.

To further elucidate the effect of sADAM10 on Aβ42, the aggregation and degradation of Aβ42 were evaluated following enzymatic treatment. Aggregation of the peptide was monitored with thioflavin T (ThT), a fluorescent dye that displays enhanced fluorescence upon binding to β-sheet-rich structures such as the Aβ fibrils (LeVine, 1999; Gade Malmos et al., 2017). Monomeric Aβ42 was incubated and treated with sADAM10 at different time points and their fluorescence was measured over time (Figures 3A–E). Control samples, 0 μM sADAM10, exhibited the characteristic ThT sigmoidal profile, signifying the assembly of the monomeric Aβ42 to fibrils (Cohen et al., 2012). Results show that simultaneous treatment of the monomeric peptide with sADAM10 completely suppressed the fibril formation (Figure 3A). Treatment at later time points (2 and 6 h), during the formation of the Aβ42 aggregates, resulted in the inhibition of further aggregation (Figures 3B, C). However, sADAM10 was unable to significantly disaggregate the already formed fibrils in solution, as exhibited with treatment at 12 and 24 h (Figures 3D, E). In comparison, treatment of the formed fibrils with plasmin, an Aβ fibril degrading enzyme (Tucker et al., 2000), resulted in a decrease in ThT fluorescence (Figure 3E).

Furthermore, the effect of sADAM10 on the hydrolysis of Aβ42 was determined. Aβ42 was treated with sADAM10, followed by analysis with HPLC to determine the formation of Aβ16, an indication of Aβ42 cleavage. When monomeric Aβ42 was treated with the enzyme, an increase in Aβ16 was measured over time (Figure 3F), supporting Aβ42 hydrolysis. The formation of Aβ16 reached a plateau 2-h post-treatment, as visible by the Aβ16 levels at 2 and 6 h. To determine the effect of the enzyme during different aggregation states, monomeric Aβ42 was first allowed to aggregate at different times. Then, sADAM10 was added for a 6-h incubation to ensure maximum detectable hydrolysis, followed by Aβ16 analysis (Figure 3G). The Aβ42 oligomers formed after a 2-h aggregation period and subsequently treated with sADAM10 resulted in almost full degradation as compared to the control group (i.e., monomeric Aβ42 following a 6-h treatment period). Samples treated after a longer aggregation period produced lower concentrations of Aβ16, suggesting that the enzyme was unable to hydrolyze Aβ42 in the solution once the latter had aggregated. Similar to the ThT aggregation results, incubation of the aggregated Aβ42 with plasmin resulted in an increase of Aβ16 confirming its ability to hydrolyze amyloid fibrils.

### 3.4. Treatment of differentiated SH-SY5Y cells with the soluble secretase inhibits Aβ42 aggregation

**Figure 4:**
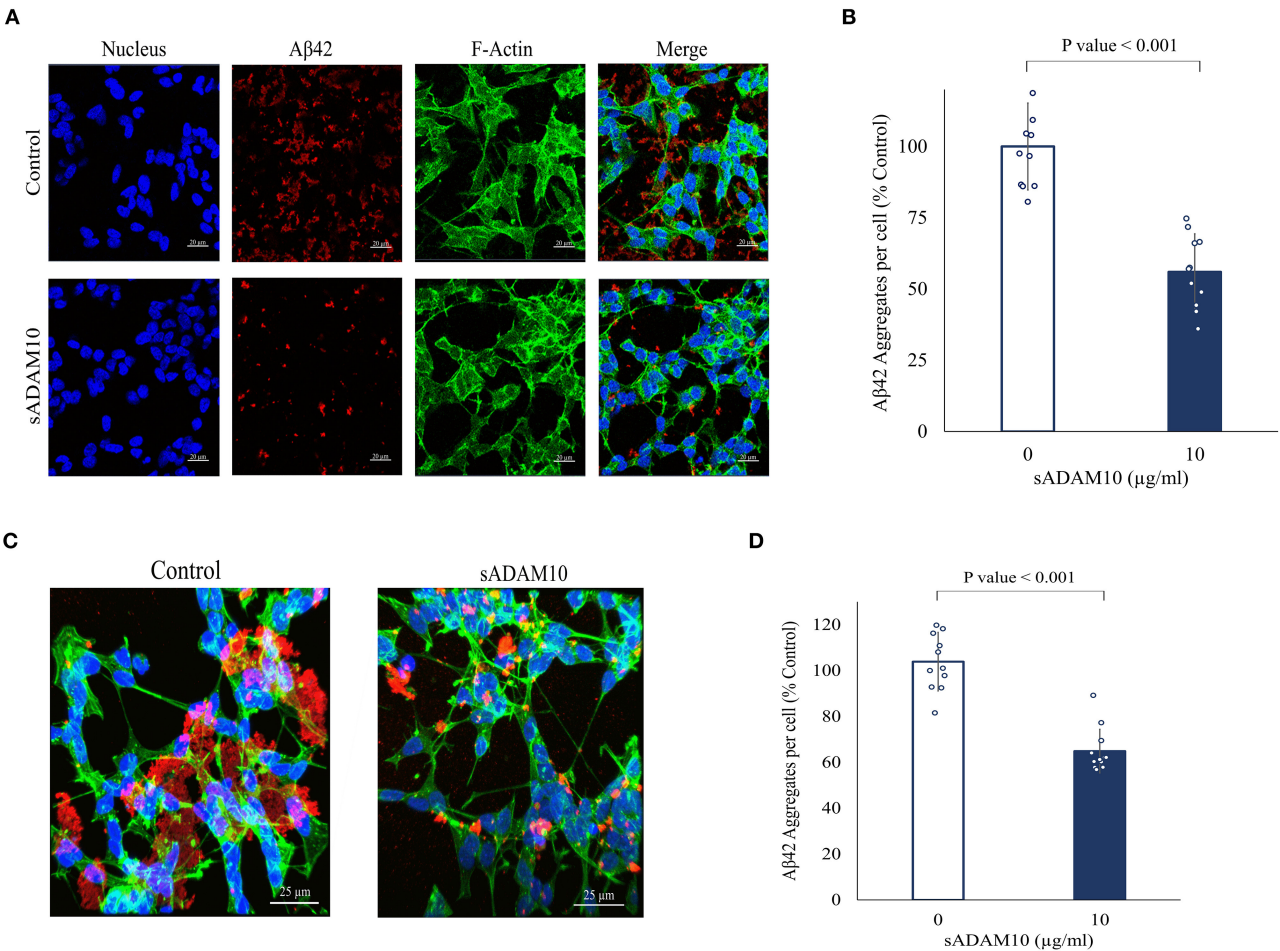
Effect of sADAM10 treatment on Aβ42 induced aggregates in differentiated SH-SY5Y cells. **(A)** Representative confocal images of cells with Aβ42 aggregates 24-h post-treatment. Control, untreated cells. ADAM10, cells were treated simultaneously with 10 μg/ml sADAM10 and monomeric Aβ42. **(B)** Aβ42 aggregates per cell of differentiated SH-SY5Y cells 24-h post-simultaneous treatment. **(C)** Representative confocal images of cells with Aβ42 aggregates 48-h post-induction. Control, untreated cells. ADAM10, cells treated with 10 μg/ml sADAM10 24-h post-aggregate induction. **(D)** Aβ42 aggregates per cell of differentiated SH-SY5Y cells 24-h post-treatment of aggregated Aβ42. Nucleus (DAPI, blue), Aβ42 (6E10, red), F-actin (phalloidin-FITC, green). Images are at × 40 magnification. Aggregate volume was quantified with confocal microscopy and Imaris software. Data are from two independent experiments performed in duplicate.

While the sADAM10 was unable to hydrolyze pure and aggregated Aβ42 in solution, it was suggested that it may prevent the formation of new aggregates from soluble peptides in a cellular environment. To that end, the ability of sADAM10 to inhibit Aβ42 aggregation in neuronal cells was determined (Figure 4). Differentiated SH-SY5Y cells were incubated simultaneously with monomeric Aβ42 to initiate aggregation and with sADAM10 as treatment. Following a 24-h incubation, the cells were fluorescently stained and imaged by confocal scanning microscopy, and Aβ42 aggregate levels per cell were determined. Treatment with the α-secretase decreased aggregate levels per cell by 50% (*p* < 0.001) compared to untreated cells (Figures 4A, B). Furthermore, to evaluate the effect of sADAM10 on already formed extracellular aggregates, monomeric Aβ42 was added to differentiate SH-SY5Y cells for 24 h to allow for the formation of aggregates prior to treatment. Subsequently, the cells' media was replaced with fresh neuronal treatment media (i.e., media with sADAM10 or PBS). Following a 24-h treatment period, the cells were imaged by confocal scanning microscopy (Figure 4C), and levels of Aβ42 aggregates per cell were analyzed (Figure 4D). The results indicate that the addition of the sADAM10 to the already formed aggregates has an inhibiting effect on the aggregation process, as seen in significantly lower aggregates per cell in the treated group (*p* < 0.001).

### 3.5. Identification of soluble ADAM10 substrates by proteomic analysis

**Figure 5:**
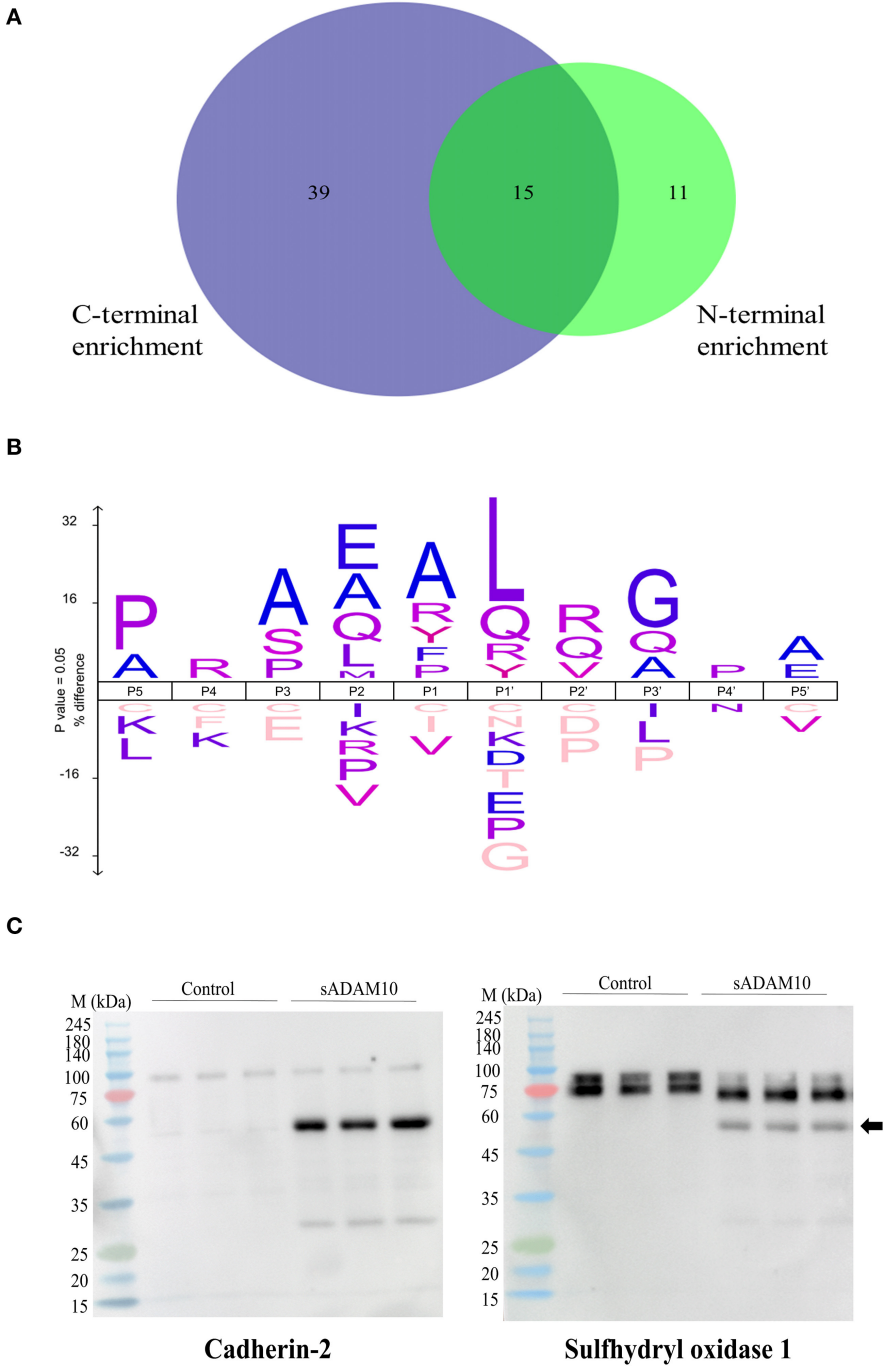
Proteomic identification of potential sADAM10 substrates in differentiated SH-SY5Y cells following N-terminal and C-terminal enrichment. **(A)** Venn Diagram of cleaved proteins with N-terminal enrichment and C-terminal enrichment. **(B)** Cleavage site sequence logo of sADAM10 mediated cleavages. **(C)** Validation of selected predicated substrates. Western blot analysis of supernatant following treatment with sADAM10. Cross-reactivity of antibody with sADAM10 is marked by a black arrow. M, molecular weight marker (kDa).

Substrates of membrane-bound ADAM10 have been well characterized and reviewed (Pruessmeyer and Ludwig, 2009; Kuhn et al., 2010; Rawlings et al., 2012; Prox et al., 2013; Tucher et al., 2014; Saftig and Lichtenthaler, 2015). Recently, a proteomic analysis of the secretome of murine cardiomyocytes demonstrated that the soluble ectodomain of ADAM10 exhibited different target specificities as compared to its membrane-bound form (Scharfenberg et al., 2020). Therefore, to further assess the cleavage capabilities of the soluble secretase, a proteomic approach was applied to evaluate the potential substrates of sADAM10 in differentiated neuronal cells. To this aim, differentiated SH-SY5Y was treated with or without sADAM10, and media was prepared and analyzed using N-terminal enrichment by HYTANE (Chen et al., 2016; Weng et al., 2019) or C-terminal enrichment. The proteomic analyses identified cleavage sites and open reading frame N-terminal or C-terminal peptides of over 1,400 proteins. To evaluate putative sADAM10 extracellular substrates, we considered only peptides derived from either membrane-bound or secreted proteins. A peptide derived from these proteins would be considered a putative sADAM10 cleavage only if it appears in the two biological repeats with an at-least three-fold change in the ratio of the sADAM10-treated sample compared to control [log_2_(sADAM10/control) > 1.6]. This resulted in 155 peptides derived from 65 proteins. The putative sADAM10 substrates and the number of identified cleavage sites are shown in Table 1. Supplementary Table 1 includes the peptides, their cleavage site motif, enrichment method, and ratio. The N-terminal enrichment displayed 26 potential substrates, while the C-terminal enrichment identified 54 potential substrates, with 15 overlapping substrates (Figure 5A).

**Table 1:** Putative sADAM10 substrates.

	**Protein**	**Gene**	**UniProt**	**Enrichment method**	**Type**	**Number of cleavage sites**	**References**
1	ADAMTS-like protein 2	ADAMTSL2	Q86TH1	C-terminal	Secreted	1	
2	Gamma-adducin	ADD3	Q9UEY8	C-terminal	Membrane	1	
3	Amyloid-beta precursor protein	APP	P05067	C-terminal	Membrane	1	Lammich et al., 1999; Postina et al., 2004; Jorissen et al., 2010; Kuhn et al., 2010
4	Calcitonin gene-related peptide 2	CALCB	P10092	C-terminal	Secreted	1	
5	Cadherin-2, N-cadherin	CDH2	P19022	C-terminal	Membrane	2	Reiss et al., 2005; Uemura et al., 2006; Scharfenberg et al., 2020
6	Cadherin EGF LAG seven-pass G-type receptor 2	CELSR2	Q9HCU4	N-terminal	Membrane	1	
7	Cofilin-1	CFL1	P23528	C and N- terminal	Membrane	4	
8	Chromogranin-A	CHGA	P10645	C and N- terminal	Secreted	19	
9	Secretogranin-1	CHGB	P05060	C and N- terminal	Secreted	10	
10	Calsyntenin-1	CLSTN1	O94985	C-terminal	Membrane	1	Hata et al., 2009; Zhou et al., 2020
11	Clathrin heavy chain 1	CLTC	Q00610	C-terminal	Membrane	1	
12	Adapter molecule crk	CRK	P46108	C-terminal	Membrane	1	
13	Cystatin-C	CST3	P01034	N-terminal	Secreted	1	Scharfenberg et al., 2020
14	Src substrate	CTTN	Q14247	C-terminal	Membrane	2	
15	Dopamine beta-hydroxylase	DBH	P09172	C-terminal	Secreted	1	
16	Drebrin	DBN1	Q16643	C-terminal	Membrane	1	
17	Protocadherin-16	DCHS1	Q96JQ0	C-terminal	Membrane	1	Kuhn et al., 2016
18	Dihydropyrimidinase-related protein 2	DPYSL2	Q16555	C and N- terminal	Membrane	2	
19	Alpha-enolase	ENO1	P06733	C and N- terminal	Membrane	5	
20	Gamma-enolase	ENO2	P09104	C-terminal	Membrane	1	
21	RNA-binding protein EWS	EWSR1	Q01844	N-terminal	Membrane	1	
22	FERM, ARHGEF, and pleckstrin domain-containing protein 1	FARP1	Q9Y4F1	C-terminal	Membrane	1	
23	Fibulin-2	FBLN2	P98095	N-terminal	Secreted	1	
24	FXYD domain-containing ion transport regulator 6	FXYD6	Q9H0Q3	C-terminal	Membrane	1	
25	Galanin peptides	GAL	P22466	N-terminal	Secreted	2	
26	Glyceraldehyde-3-phosphate dehydrogenase	GAPDH	P04406	C and N- terminal	Membrane	12	
27	Glycine–tRNA ligase	GARS1	P41250	C-terminal	Secreted	1	
28	Growth/differentiation factor 10 (GDF-10)	GDF10	P55107	N-terminal	Secreted	1	
29	Hepatoma-derived growth factor	HDGF	P51858	C-terminal	Secreted	1	
30	Hepatocyte growth factor-regulated tyrosine kinase substrate	HGS	O14964	C-terminal	Membrane	1	
31	High mobility group protein B2	HMGB2	P26583	C-terminal	Secreted	1	
32	Heat shock protein HSP 90-alpha	HSP90AA1	P07900	C and N- terminal	Membrane	3	
33	Heat shock protein HSP 90-beta	HSP90AB1	P08238	C and N- terminal	Secreted, Membrane	5	
34	Heat shock cognate 71 kDa protein	HSPA8	P11142	C and N- terminal	Membrane	3	
35	KH domain-containing, RNA-binding, signal transduction-associated protein 1	KHDRBS1	Q07666	C-terminal	Membrane	3	
36	Galectin-3-binding protein	LGALS3BP	Q08380	N-terminal	Secreted	1	
37	Myristoylated alanine-rich C-kinase substrate	MARCKS	P29966	C and N- terminal	Membrane	2	
38	Protein MENT (methylated in normal thymocytes protein)	MENT	Q9BUN1	C-terminal	Secreted	1	
39	Matrix metalloproteinase-15	MMP15	P51511	N-terminal	Membrane	1	
40	Serine/threonine-protein kinase PAK 2	PAK2	Q13177	C-terminal	Membrane	1	
41	ProSAAS	PCSK1N	Q9UHG2	C and N- terminal	Secreted	2	
42	Programmed cell death 6-interacting protein	PDCD6IP	Q8WUM4	C-terminal	Secreted	1	
43	Peptidyl-prolyl cis-trans isomerase A	PPIA	P62937	C and N- terminal	Secreted	4	
44	Serine/threonine-protein phosphatase 2A 65 kDa regulatory subunit A alpha isoform	PPP2R1A	P30153	C-terminal	Membrane	1	
45	Proline-rich transmembrane protein 3	PRRT3	Q5FWE3	C-terminal	Membrane	1	
46	Sulfhydryl oxidase 1	QSOX1	O00391	C-terminal	Secreted	2	
47	Rac GTPase-activating protein 1	RACGAP1	Q9H0H5	C-terminal	Membrane	1	
48	Transforming protein RhoA	RHOA	P61586	C-terminal	Membrane	1	
49	40S ribosomal protein S8	RPS8	P62241	N-terminal	Membrane	1	
50	40S ribosomal protein SA	RPSA	P08865	C-terminal	Membrane	3	
51	Reticulon-4	RTN4	Q9NQC3	N-terminal	Membrane	1	Kuhn et al., 2016
52	Protein RUFY3	RUFY3	Q7L099	C-terminal	Membrane	1	
53	Secretogranin-2	SCG2	P13521	C and N- terminal	Secreted	2	
54	Secretogranin-3	SCG3	Q8WXD2	N-terminal	Secreted	1	
55	Neuroendocrine protein 7B2	SCG5	P05408	C-terminal	Secreted	1	
56	Septin-2	SEPTIN2	Q15019	C-terminal	Membrane	1	
57	Protein SOGA3	SOGA3	Q5TF21	C-terminal	Membrane	1	
58	C-Jun-amino-terminal kinase-interacting protein 4	SPAG9	O60271	C-terminal	Membrane	1	
59	Spectrin beta chain, non-erythrocytic 1	SPTBN1	Q01082	C-terminal	Membrane	1	
60	Proto-oncogene tyrosine-protein kinase Src	SRC	P12931	C-terminal	Membrane	1	
61	Symplekin	SYMPK	Q92797	C-terminal	Membrane	1	
62	Talin-1	TLN1	Q9Y490	C and N- terminal	Membrane	4	
63	Neurosecretory protein VGF	VGF	O15240	C and N- terminal	Secreted	20	
64	Wiskott-Aldrich syndrome protein family member 2	WASF2	Q9Y6W5	C-terminal	Membrane	1	
65	Y-box-binding protein 1	YBX1	P67809	C-terminal	Secreted	1	

The table details proteins with significant cleavage ratios of sADAM10-treated samples compared to the control samples.

Among the sADAM10 substrate candidates, previously well-described membrane-bound neuronal ADAM10 substrates were identified [e.g. APP (Lammich et al., 1999; Jorissen et al., 2010; Kuhn et al., 2010), N-cadherin (Reiss et al., 2005; Uemura et al., 2006), Calsyntenin-1 (Hata et al., 2009)]. Reticulon-4, previously identified in a 2016 proteomic analysis of ADAM10-deficient neurons (Kuhn et al., 2016), was also identified as a substrate. With regard to APP, the proteomic data also indicated a potential cleavage site in APP, different from the known secretase site (K612.L613). Recently, two other cleavage sites of sADAM10 were also recognized in APP (Scharfenberg et al., 2020). Some examples of additional substrates identified, that have not been previously reported to our knowledge, are sulfhydryl oxidase 1 (QSOX1), matrix metalloproteinase-15 (MMP15), and protocadherin-16 (DCHS1). However, closely related substrates were previously identified as membrane-bound ADAM10 substrates: QSOX2, MMP17, and other Protocadherin proteins (e.g., Protocadherin 8, 9 and 20) (Reiss et al., 2006; Bouillot et al., 2011; Kuhn et al., 2016; Pancho et al., 2020). The reported substrates above are associated with membrane-bound ADAM10. Recently, the substrate spectrum of the soluble ectodomains of ADAM10 was compared to the membrane-bound counterparts utilizing the N-terminomics approach. Similar to our results, N-cadherin and Cystatin-C were also identified as soluble ADAM10 substrates (Scharfenberg et al., 2020). In addition to the known and related substrates described above, several other proteins known to interact with ADAM10 were identified as substrates. According to the harmonizome database, a collection of processed datasets of genes and proteins, several of the proteins detected are part of the ADAM10 protein–protein interaction dataset but are not known to be direct substrates of ADAM10 [e.g., HGS, CLTC (Marcello et al., 2013) and TLN1 (Rouillard et al., 2016)]. The neurosecretory protein VGF is an example of a secreted protein significantly cleaved upon treatment by sADAM10. In addition, newly identified secreted neuronal substrates are from the chromogranin–secretogranin family [e.g., chromogranin-A (CHGA), secretogranin-1 (CHGB), secretogranin-2 (SCG2), secretogranin-3 (SCG3), and neuroendocrine protein 7B2 (SCG5)].

The identified peptides from the sADAM10 cleaved proteins from both enrichment methods were used to determine the cleavage specificity of the soluble secretase (Figure 5B). The specificity profiles from the N-terminal enrichment and C-terminal enrichment are shown in Supplementary Figures 1A, B, respectively. The identified cleavage sequence is comparable to reported cleavage sites (Caescu et al., 2009; Zhou et al., 2020), particularly it bares similarity to the recently reported cleavage site obtained via proteomic analysis of soluble ectodomain of murine ADAM10 (Scharfenberg et al., 2020).

### 3.6. Validation of sADAM10 substrates by immunoblots

Substrates from the MS-based proteomic data were chosen for validation by Western blot analysis (Figure 5C). Fragments of the known membrane-bound substrate, N-Cadherin, were identified in both the control and treated groups, with additional fragments identified in the latter, indicating cleavage by the sADAM10. A secreted protein, sulfhydryl oxidase 1, was also validated by the Western blot. Media of control cells presents two isoforms with a molecular weight above 75 kDa. Meanwhile, treated samples present a main band with a lower molecular weight, indicating cleavage of the protein at its identified cleavage site. sADAM10 was also visualized with the relevant validation antibodies as a control (Supplementary Figure 1C).

### 3.7. A truncated sADAM10 expressed in *E. coli* has APP-shedding activity

**Figure 6:**
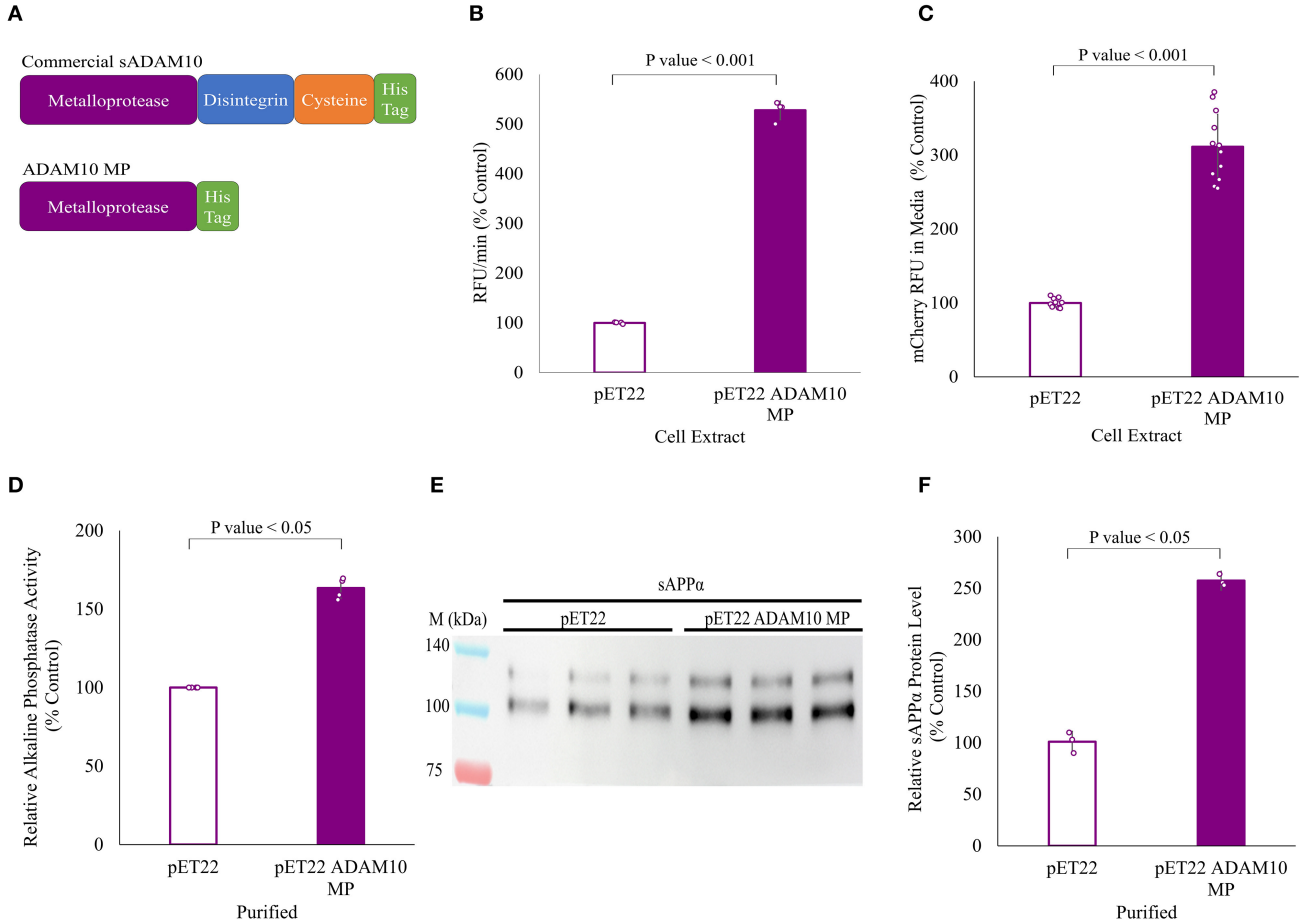
APP shedding of truncated sADAM10 MP produced from *Escherichia coli* periplasm. **(A)** sADAM10 variants used in this study. **(B)** Hydrolysis of a model fluorescent peptide by *E. coli* ADAM10 MP cell extracts. **(C)** Fluorescent soluble APP in the media of transfected HEK293T ADAM10 knockout cells following treatment with *E. coli* ADAM10 MP cell extracts. **(D)** Relative alkaline phosphatase activity of transfected HEK293T ADAM10 knockout cells following treatment with purified ADAM10 MP samples. **(E)** Representative Western blot analysis image of endogenous sAPPα levels in the media of differentiated SH-SY5Y cells following treatment with purified ADAM10 MP samples. **(F)** Western blot quantification of relative sAPPα levels with purified samples.

All of the above work was carried out with a commercial enzyme expressed in *Spodoptera frugiperda Sf* 21 insect cells. Here, the shedding capabilities of a truncated variant of the soluble ADAM10, expressed in the periplasm of *E. coli* cells, were evaluated (Figure 6). Expression in the *E. coli* cytoplasm was unsuccessful. The truncated variant, comprised solely of the ADAM10 metalloprotease catalytic domain, was designated ADAM10 MP (Figure 6A). As the truncated variant is without its regulatory domains (Seegar et al., 2017), we first sought to determine its α-secretase shedding capacity prior to purification. Periplasm extracts of bacteria expressing ADAM10 MP were compared to extracts of negative control, not expressing the enzyme. The ADAM10 MP presented a five-fold increase in catalytic activity when applied to a commercial fluorescently labeled peptide substrate (Figure 6B). Furthermore, a three-fold increase was measured in media fluorescence when the extracts were applied to ADAM10 knockout cells overexpressing the APP fluorescent fusion protein (Figure 6C).

Following expression, ADAM10 MP and its control were purified using anion exchange and nickel affinity chromatography (Supplementary Figure 2). When enzymatic shedding by the purified samples was assessed with the ADAM10 knockout cells expressing the APP alkaline phosphatase fusion protein, a 60% increase in relative alkaline phosphatase activity was obtained (Figure 6D). Finally, the shedding of endogenous APP from differentiated SH-SY5Y cells with the purified samples, resulting in a 2.5-fold increase of relative sAPPα levels when the ADAM10 MP sample was applied (Figures 6E, F). To our knowledge, this is the first time a soluble ADAM10 MP variant was successfully expressed in *E. coli* cells in active form.

## 4. Discussion

The Aβ hypothesis, proposed over 30 years ago, has been at the core of the AD drug development pipeline. Potential treatments have focused on targeting Aβ production, its pathway, and clearing the amyloid deposits by means of antibodies and enzymatic inhibitors. However, most drug candidates failed to demonstrate clinical efficacy, most likely due to late therapeutic intervention, as the underlying disease pathology begins decades before AD diagnosis (Jack et al., 2013). The recent clinical trials focusing on AD patients in early clinical stages indicate that intervention is necessary at the early stages of the disease. The emerging treatments targeting disease modifications such as the Aβ pathway may be beneficial to improve AD treatment outcomes (Tolar et al., 2020b; Hampel et al., 2021; Karran and de Strooper, 2022; Levin et al., 2022). Recently, a few anti Aβ drugs for the treatment of AD were approved by the FDA (Cavazzoni, 2021; Cummings et al., 2022). These potential disease-modifying treatments are antibodies characterized by their ability to target toxic Aβ oligomers and plaques, thus stimulating the immune system to reduce their levels (Sevigny et al., 2016; Tolar et al., 2020a; Mintun et al., 2021; Swanson et al., 2021). Clinical results of the drug candidates also demonstrate that intervention in the early stages of AD is essential. Furthermore, they highlight the necessity for the development of new effective anti Aβ therapies. Here, we propose a new enzymatic approach for the treatment of AD, namely, to promote the non-amyloidogenic pathway and degrade the neurotoxic Aβ using a soluble form of ADAM10. We demonstrated that sADAM10 can shed APP from cells, generating the non-amyloidogenic pathway product, sAPPα. Additionally, our results indicate that sADAM10 can inhibit the Aβ aggregation process. In addition, we developed a novel truncated sADAM10 comprising the metalloprotease domain only and demonstrated its capability of releasing sAPPα. Increasing the enzymatic activity of ADAM10 necessitates a better understanding of the soluble form's substrates and side effects. Consequently, we identified potential substrates using a proteomics approach enriching N and C-terminal peptides of cleavage fragments.

Therapeutic targeting of ADAM10 in AD is considered advantageous as it is the main secretase involved in the processing of APP in the non-amyloidogenic pathway. Moreover, it has been directly linked to the pathogenesis of AD (Endres and Fahrenholz, 2010; Prox et al., 2013; Marcello et al., 2017; Musardo and Marcello, 2017). Mutations identified in the ADAM10 pro-domain resulted in lower enzymatic activity and accentuated the amyloidogenic pathway increasing the Aβ aggregates (Kim et al., 2009; Suh et al., 2013). Furthermore, genome-wide association studies identified the ADAM10 gene as a risk factor for AD (Jansen et al., 2019; Kunkle et al., 2019). More recently, a clinical and genetic study of a family with AD identified a mutation in the ADAM10 pro-domain. This was found to be correlated with lower levels of APP processing in the non-amyloidogenic pathway as evidenced by reduced sAPPα (Agüero et al., 2020). In a longitudinal study of older adults, low levels of active ADAM10 correlated with a decline in cognitive abilities (Oliveira Monteiro et al., 2021). Moreover, ADAM10 is interrelated to the disease as levels of ADAM10 are reduced in AD patients (Colciaghi et al., 2002, 2004).

Considering the ADAM10 relevance to AD, research has focused on increasing its activity and promoting the non-amyloidogenic pathway *in vitro* and in a clinical setting (Postina et al., 2004). For example, enzymatic activity was upregulated in an *in vivo* study by interfering with ADAM10 endocytosis (Musardo et al., 2022). Other studies focus on repurposing FDA-approved drugs, for instance, research into disulfiram as an ADAM10 gene enhancer resulted in lower Aβ aggregates and an increase in functional behavior tests in mice (Reinhardt et al., 2018). Acitretin, a psoriasis medication, is another extensively studied repurposed drug for AD capable of inducing ADAM10 activity *in vivo* and clinically (Tippmann et al., 2009; Endres et al., 2014; Brummer et al., 2019; Rosales Jubal et al., 2021). Similarly, a phase 2 clinical trial drug candidate, APH-1105, is said to modulate α-secretase activity by activating protein kinase C (Cummings et al., 2022).

In support of this line of thought, we sought to increase the non-amyloidogenic pathway via exogenous enzymatic treatment with a soluble form of human ADAM10, specifically with its catalytic domain. ADAM10 MP isolated from the periplasm is the first soluble ADAM10 protein expressed in *E. coli* to present α-secretase shedding activity. Previous research purified the pro-domain of murine ADAM10 from *E. coli* to investigate its specific inhibition of ADAM10 (Moss et al., 2007). The ADAM10 ectodomain was successfully expressed in insect cells using the baculovirus system, and its crystal structure was determined (Seegar et al., 2017). More recently, soluble ectodomains of murine ADAM10 and ADAM17, an α-secretase with a similar structure and functions to ADAM10 (Edwards et al., 2008), were also purified in insect cells for proteomic substrate identification (Scharfenberg et al., 2020). The sADAM10 variants tested shed APP from the cell surface by cleavage at the α-secretase site, preventing the first event of the amyloidogenic pathway required for the formation of Aβ peptides and releasing sAPPα above its endogenous level. However, the increase in endogenous sAPPα could also potentially be due to an increase in APP expression emanating from the enzymatic treatment. This cannot be inferred from the study and future research on the sADAM10 effect on APP expression is required. Notwithstanding, both tested sADAM10 variants, with and without the regulatory domains [i.e., disintegrin and cysteine-rich domains (Seegar et al., 2017)], were able to release sAPPα from neuronal cultures. An increase in sAPPα would exploit its natural neuroprotective and neurotrophic effects as part of the AD treatment. Demonstrated sAPPα therapeutic properties include synaptogenesis, modulation of spine density, memory enhancement, and stimulation of adult neurogenesis (as reviewed in Mockett et al., 2017; Dar and Glazner, 2020). For instance, overexpression of sAPPα in an *in vivo* model resulted in improved synaptic plasticity, cognitive function, and reduced levels of Aβ (Fol et al., 2016). Elevated sAPPα levels in AD would also decrease the processing of APP via the amyloidogenic pathway by allosteric inhibition of BACE1, the β-secretase responsible for the generation of Aβ (Obregon et al., 2012; Peters-Libeu et al., 2015). Consequently, this would also prevent downstream events in AD progression, such as Aβ-induced dendritic spine loss and tau phosphorylation (Deng et al., 2015; Tackenberg and Nitsch, 2019).

In addition to inhibiting the synthesis of Aβ peptides, as the truncated enzyme is not cell-bound, it also decreases the neurotoxic Aβ by directly targeting the peptide at different aggregation steps. Extensive research has shown that Aβ oligomers are the primary neurotoxic species implicated in advancing AD pathogenesis (Kayed et al., 2003; Nimmrich et al., 2008; He et al., 2012; Hayden and Teplow, 2013; Shea et al., 2019; Uhlmann et al., 2020; Hampel et al., 2021). Evidence from recent clinical trials with anti-amyloid antibodies indicates that targeting Aβ oligomers are clinically preferential (Tolar et al., 2021). We demonstrated that the addition of sADAM10 to monomeric and oligomeric Aβ species resulted in Aβ degradation thus preventing the aggregation process. Moreover, sADAM10 impeded the aggregation process of existent neuronal extracellular Aβ aggregates. The benefit of such a degrading enzyme is its ability to inhibit the neurotoxic Aβ aggregation process as well as to degrade the accumulating Aβ monomers, which could potentially result in hindering AD pathological progression (Miners et al., 2011; Chen et al., 2017; Sikanyika et al., 2019). Altogether, these results strengthen the ability of sADAM10 to shift the balance of APP processing toward the non-amyloidogenic pathway.

As mentioned above, ADAM10 plays a critical role in the pathology of AD, and as it has a wide range of substrates, it is also an integral part of health and disease (Pruessmeyer and Ludwig, 2009; Saftig and Lichtenthaler, 2015; Kuhn et al., 2016; Wetzel et al., 2017; Smith et al., 2020). An increase in its activity could promote proliferation and cell migration. For example, overexpression of ADAM10 is associated with cancer, most likely as a result of its part in Notch signaling (Guo et al., 2012; Mullooly et al., 2015). Therefore, its use as a therapeutic target for AD needs to be assessed with regard to potential side effects. To this end, we utilized a proteomic approach to evaluate the potential substrates of the sADAM10 on membrane-bound and secreted proteins of a neuronal cell line. By employing terminomics (Chen et al., 2016; Weng et al., 2019), we identified 65 potential sADAM10 substrates (Figure 5A and Table 1). Most of the terminomics approaches for protease substrate discovery are based on the enrichment of N-terminal peptides. These methods were proven to provide essential biological information that would have been difficult to obtain otherwise (Marino et al., 2015). However, they do have some limitations that restrict the identification of potential of N-terminal peptides including the identification of neo-N-terminal peptides that were generated after proteolytic processing. To overcome this and expand the characterization of sADAM10 putative cleavage sites, we combined N-terminal enrichment (Chen et al., 2016; Weng et al., 2019) and C-terminal enrichment (Hanna et al., 2023). This combined approach provides a more comprehensive view of the putative sADAM10 substrates, as demonstrated for other proteases (van Damme et al., 2010). In particular, the addition of C-terminal enrichment proved extremely useful for identifying neo-C-terminal peptides generated from membrane proteins shed after ADAM10 activity.

The results show that sADAM10 sheds several known ADAM10 membrane-bound substrates as well as additional secreted ones. Four previously established membrane-bound substrates were also identified as sADAM10 substrates (i.e., N-cadherin, calsyntenin-1, APP, and reticulon-4). Moreover, the proteomic data indicated a new cleavage site in the known membrane-bound substrate APP. Similarly, a proteomic study of murine-soluble ADAM10 in the secretome of murine cardiomyocytes revealed additional cleavage sites in APP (Scharfenberg et al., 2020). Similarly, the proteomic study of murine-soluble ADAM10 also identified cystatin-C, a secreted protein, as a substrate (Scharfenberg et al., 2020).

Another previously identified substrate is the membrane-bound reticulon-4, which functions as a negative regulator of neuronal growth in the central nervous system (Kuhn et al., 2016). With regard to AD, the expression of reticulon-4 impairs neurite and axonal growth (Schwab, 2010). Another interesting substrate regarding AD is the secreted protein VGF that has yet to be reported as an ADAM10 substrate. VGF and peptides derived from its processing play many roles in neurogenesis and neuroplasticity associated with learning, memory, depression, and chronic pain (Lewis et al., 2015). In AD pathogenesis, VGF was highlighted as a potential therapeutic target as the protein, and its active peptides were reported to reduce amyloid plaques and decrease neuritic dystrophy (el Gaamouch et al., 2020; Quinn et al., 2021). It has also been identified as a key regulator of AD since its levels are reduced in Alzheimer's patients (Duits et al., 2018; Beckmann et al., 2020). An additional newly identified secreted substrate is neuroendocrine protein 7B2, which functions as a chaperone for the prohormone convertase PC2 protein, a key activator of prohormones and neuropeptide precursors within the regulated secretory pathway of neuroendocrine cells (Mbikay et al., 2001; Helwig et al., 2013). Moreover, hepatocyte growth factor-regulated tyrosine kinase (HGS) is a newly identified membrane-bound substrate. HGS and its receptor tyrosine kinase MET have a role in uncontrolled cell survival, growth, and angiogenesis in cancer. One of the mechanisms that lead to the downregulation of MET is shedding by ADAM10 (Gherardi et al., 2012). Overall, this proteomic study highlights the potential effects that sADAM10 could have on different mechanisms and pathways. The newly identified substrates are a starting point for future research into the influence of the treatment with exogenous sADAM10 and its truncated variant.

The development of new therapies targeting the early preclinical stage with the ability to slow the disease progression is crucial. The focus of our study was to develop an enzyme based on the catalytic domain of ADAM10 for the potential treatment of AD. Exogenous treatment with a soluble variant of ADAM10 would work in parallel mechanisms. sADAM10 targeted toward APP would increase the α-secretase activity, directly decreasing the amyloidogenic pathway by competing with endogenous β-secretase for APP as a substrate (Postina et al., 2004), thus shifting the balance toward the non-amyloidogenic pathway. This would result in the release of neuroprotective sAPPα and inhibit Aβ synthesis. Furthermore, sADAM10 would degrade the main neurotoxic Aβ component in the extracellular aggregates and inhibit their formation. However, an increase in α-secretase activity with a soluble ADAM10 variant warrants further research regarding consequential side effects. Truncated variants that lack the disintegrin and cysteine-rich regulatory domains, may prove to be more selective in that the enzyme will not bind to various substrates which require such interactions. However, the effect of sADAM10 truncation on cleavage specificity remains to be assessed using a proteomic approach or *in vivo* studies (Atapattu et al., 2016; Seegar et al., 2017). Potential AD treatment with a recombinant truncated ADAM10 that would target an early stage in the cellular events and address the underlying disease has not yet been reported. Further development of the expression and purification process is required due to low solubility and concentration yields, as well as the assessment of its efficacy as a novel AD treatment *in vivo*. Additionally, enzymatic activity modulation via protein engineering is suggested, thus improving its selectivity toward APP while reducing activity toward other substrates.

## Data availability statement

The mass spectrometry proteomics data have been deposited to the ProteomeXchange Consortium via the PRIDE [1] partner repository with the dataset identifier PXD040511.

## Author contributions

AH and SG conducted the data preprocessing, analysis, and contributed to the design of the study. RH researched the proteomic data. OK contributed to the design of the proteomic analysis and validated the proteomic data. AS contributed to the conception and design of the study. AH wrote the first draft of the manuscript. AF supervised, contributed to the conception and design of the study, reviewed, and edited the manuscript. All authors contributed to the article, reviewed the manuscript, and approved the submitted version.
