# Supplementary material for: Shifting the balance: soluble ADAM10 as a potential treatment for Alzheimer's disease
Source: Front Aging Neurosci. 2023 May 17;15:1171123. doi: 10.3389/fnagi.2023.1171123 (PMC10229884; doi:10.3389/fnagi.2023.1171123)
Supplement: Supplementary file 1 [file Data_Sheet_1.DOCX]

Supplementary Material

**Shifting the balance: Soluble ADAM10 as a potential treatment for Alzheimer’s disease**

**Ayelet Sarah Hershkovits^1,2^** ^†^**, Sivan Gelley^1^** ^†^**, Rawad Hanna^3^, Oded Kleifeld^3^, Avidor Shulman^4^ and Ayelet Fishman^1^ ***

*Corresponding author: Ayelet Fishman, [afishman@technion.ac.il](mailto:afishman@technion.ac.il)


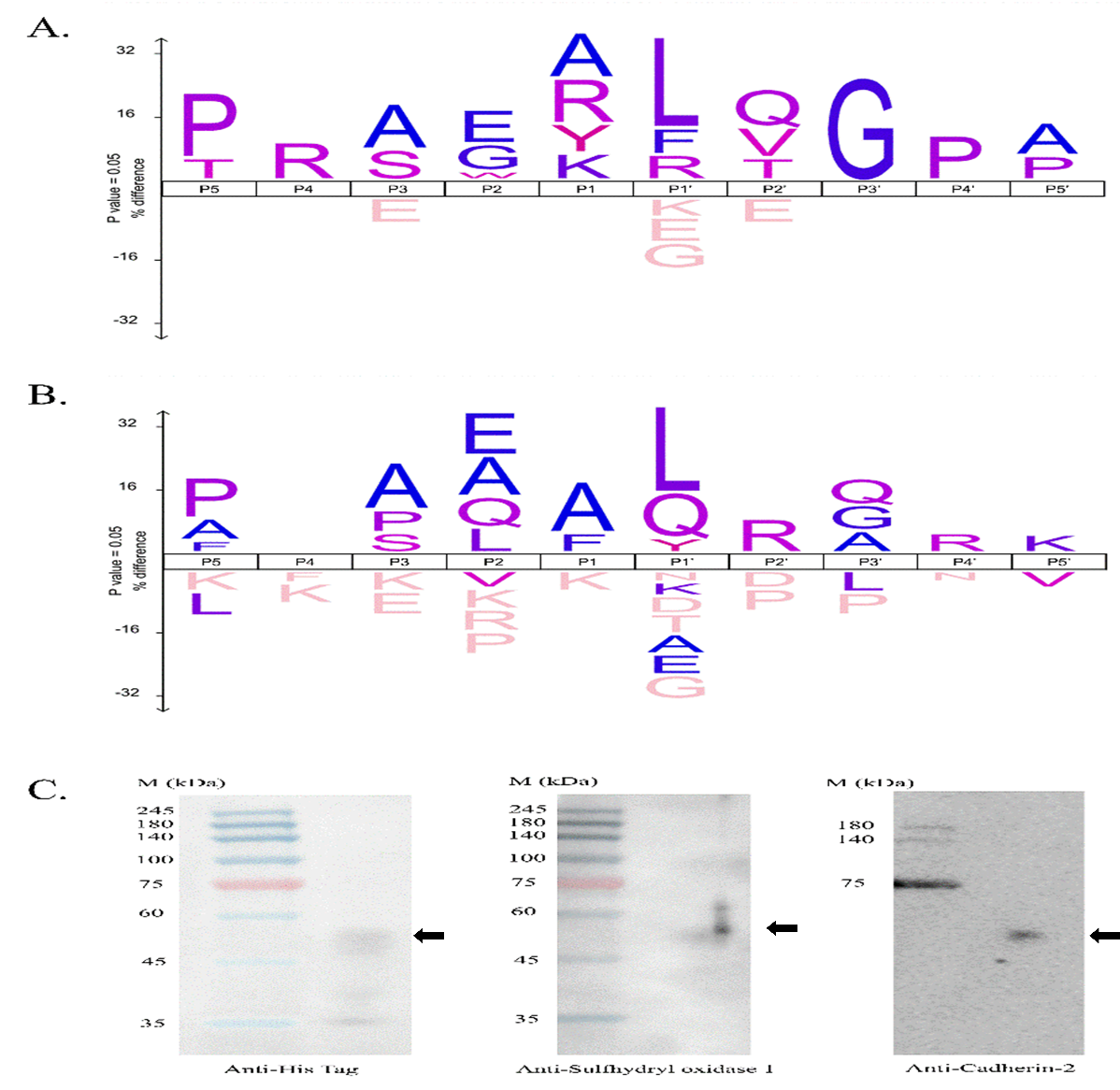


**Supplementary Figure 1** A. N- terminal enrichment cleavage site sequence logo of sADAM10 mediated cleavages B. C- terminal enrichment cleavage site sequence logo of sADAM10 mediated cleavages. Cleavage site sequence logo was generated with IceLogo C. Western blot analysis of sADAM10 with validated substrate antibodies. Anti-His tag for sADAM10 identification. sADAM10 and its cross reactivity is marked by black arrow. M: molecular weight marker (kDa)
