# Supplementary material for: Shifting the balance: soluble ADAM10 as a potential treatment for Alzheimer's disease
Source: Front Aging Neurosci. 2023 May 17;15:1171123. doi: 10.3389/fnagi.2023.1171123 (PMC10229884; doi:10.3389/fnagi.2023.1171123)
Supplement: Supplementary file 2 [file Data_Sheet_2.DOCX]

Supplementary Material

**Shifting the balance: Soluble ADAM10 as a potential treatment for Alzheimer’s disease**

**Ayelet Sarah Hershkovits^1,2^** ^†^**, Sivan Gelley^1^** ^†^**, Rawad Hanna^3^, Oded Kleifeld^3^, Avidor Shulman^4^ and Ayelet Fishman^1^ ***

*Corresponding author: Ayelet Fishman, [afishman@technion.ac.il](mailto:afishman@technion.ac.il)





**Supplementary Figure 2** Purification process of ADAM-10 MP and its control from *E. coli* periplasm**.** A and B – anion exchange purification Coomassie staining (A) and anti His-tag western blot (B), C and D – nickel affinity purification Coomassie staining (C) and anti His-tag western blot (D). Acronyms in A and B: CE – soluble cell extract, FT – flow-through, E – 1M NaCl. Acronyms in C and D: L – load (flow-through from second anion exchange purification step, FT – flow-though, W – wash, E1 – 50mM Imidazole, and E2 – 500mM Imidazole. Red arrowheads point at the expected size of ADAM10_MP. Gels: 15% acrylamide, load: CE, load and FT – 25µg by Bradford, W, E1 and E2 – 30µL.
